# Analyses of Exosomal HER2 in Breast Cancer and the Effect of Respective Exosome-Immune Complexes on Trastuzumab-Based Immunotherapy

**DOI:** 10.3390/ijms262110331

**Published:** 2025-10-23

**Authors:** Jordan Gorospe, Marjorie Shapiro, Venkateswara R. Simhadri

**Affiliations:** Division of Product Quality Research IV, Office of Pharmaceutical Quality and Research, Center for Drug Evaluation Research, Food and Drug Administration, Silver Spring, MD 20993, USA

**Keywords:** exosomes, HER2, trastuzumab, breast cancer, tumor microenvironment, immunotherapy, ADCC, SP-IRIS, biomarkers

## Abstract

Monoclonal antibodies like trastuzumab have shown clinical success in cancer treatment, but patient responses vary, and resistance can develop, possibly due to tumor microenvironment factors. In this study, we explored the role of HER2-positive exosomes in counteracting one of the mechanisms of action of trastuzumab: antibody-dependent cell-mediated cytotoxicity (ADCC). We conducted a comprehensive analysis of HER2 expression on exosomes purified from the plasma of breast cancer patients and different breast cancer cell lines using various purification methods. Purified exosomes were analyzed using the single-particle interferometric reflectance imaging sensor (SP-IRIS)-based ExoView platform. To gain better insight into the formation of exosomal-immune complexes with trastuzumab, we used the ExoView platform to analyze the CD9/HER2/Human IgG phenotype of exosomes at the single vesicle level. Additionally, in a standard functional ADCC assay, formation of exosome-immune complexes with trastuzumab reduced the killing of breast cancer target cells. Together, our findings show that exosomes can function as decoys for immunotherapy, reducing its efficacy, and that SP-IRIS-based analysis can be used to identify levels of HER2-expressing exosomes in patients, which could aid in patient management.

## 1. Introduction

Targeting the surface molecules expressed on malignant cells has been a hallmark of tumor immunotherapy and was clinically proven efficacious by one of the earliest monoclonal antibodies, trastuzumab, against Human Epidermal Growth Factor Receptor 2 (HER2) in breast cancer [1,2,3]. Although the success improved the survival of patients, the response to immunotherapy varies, with some patients not responding or acquiring resistance to trastuzumab [4,5]. The tumor microenvironment (TME) can play a role in how a patient responds to certain drugs. Exosomes are small extracellular vesicles, ranging from 50–200 nm in diameter, that play crucial roles in intercellular communication and disease progression [6,7]. These vesicles, once considered mere cellular waste, are now recognized as key mediators in various biological processes, including immune regulation, apoptosis, and metastasis [8]. Exosomes are formed through a complex biogenesis pathway involving the endosomal system and carry a diverse cargo of proteins, lipids, and nucleic acids, reflecting their cell of origin [9]. In cancer, tumor-derived exosomes (TDEs) contribute significantly to tumorigenesis and metastasis through multiple mechanisms [10,11]. These include the transfer of oncogenic material, stimulation of pro-inflammatory factors, recruitment of immunosuppressive cells, and modulation of the tumor microenvironment [8,10,12]. TDEs can also facilitate epithelial-to-mesenchymal transition (EMT) and promote angiogenesis, further supporting tumor progression and metastatic spread [13]. Of particular interest is the role of exosomes in HER2-positive breast cancer, a subtype affecting 20–25% of breast cancer patients and associated with poor prognosis [4]. The advent of monoclonal antibody therapy, specifically trastuzumab, revolutionized the treatment of HER2-positive breast cancer by providing a targeted approach with enhanced efficacy and reduced toxicity compared to traditional therapies [14,15]. Trastuzumab, a monoclonal antibody therapy for HER2-positive breast cancer, operates through multiple mechanisms of action. Trastuzumab inhibits HER2 receptor dimerization and signaling, disrupting downstream pathways critical for cell proliferation and survival, such as PI3K/AKT and MAPK [16,17]. The antibody also induces receptor downregulation by promoting HER2 internalization and degradation through ubiquitination and lysosomal processes [18,19]. In addition, trastuzumab also impacts cell cycle progression, downregulating proteins like RAD51, leading to DNA damage accumulation and G1 phase arrest [20,21]. Furthermore, trastuzumab stimulates antibody-dependent cellular cytotoxicity (ADCC) by binding to HER2 on tumor cells and activating immune effector cells, primarily natural killer (NK) cells, through Fc region interactions. This ADCC activation results in the release of cytotoxic molecules, enhancing anti-tumor immune responses [22,23]. These diverse mechanisms collectively contribute to trastuzumab’s efficacy in treating HER2-positive breast cancers. Recent studies have highlighted the potential impact of exosomal immune complexes on the efficacy of therapeutic antibodies [24]. However, existing methods for studying these complexes, such as electron microscopy, flow cytometry, and Western blotting, often lacked the resolution or specificity to analyze individual particles and their associated markers [25]. This resolution or specificity limitation of the tests has hindered our understanding of the heterogeneity and functional implications of exosomal-immune complexes in cancer therapy. To address these limitations, we employed Single-Particle Interferometric Reflectance Imaging Sensor (SP-IRIS) technology, which enables high-resolution, single-particle analysis of exosomes. SP-IRIS combines antibody-specific capture chips with fluorescence-based detection, allowing for the visualization and quantification of individual exosomes and their associated markers with high specificity. This innovative approach offers several advantages, including single-particle resolution, multiplexed marker detection, quantitative analysis, minimal sample processing, suitable sensitivity, and size discrimination. SP-IRIS can detect exosomes at adequately low concentrations and can distinguish exosomes from other extracellular vesicles based on size [26]. By leveraging these capabilities, SP-IRIS enables a more comprehensive analysis of exosomal-immune complexes than previously possible and forms the basis of this study investigating the interactions between exosomes, therapeutic antibodies, and their potential impact on treatment efficacy in HER2-positive breast cancer patients. The present study aimed to characterize tumor-derived exosomes (TDEs) from both cell culture media and patient plasma using SP-IRIS technology, with a focus on HER2-positive breast cancer. We investigated marker colocalization patterns and specific binding to therapeutic antibodies, particularly trastuzumab. Additionally, we developed complementary functional assays, including ADCC and cell binding assays, to assess the biological effects of exosomal immune complexes on trastuzumab’s efficacy. By combining SP-IRIS with these functional assays, we sought to establish a comprehensive platform for studying exosomal-immune complexes and their potential implications for therapeutic efficacy. This research was designed to increase understanding of potential trastuzumab resistance mechanisms and to find additional biomarkers for HER2-positive breast cancer, and could provide added insight into treatments of other malignancies. Furthermore, we anticipate that the insights gained from this study could inform therapeutic strategies that account for the influence of exosomal immune complexes on antibody-based treatments.

## 2. Results

### 2.1. Detection of HER2-Positive Particles from Breast Cancer Cell Lines Using SP-IRIS

**Figure 1:**
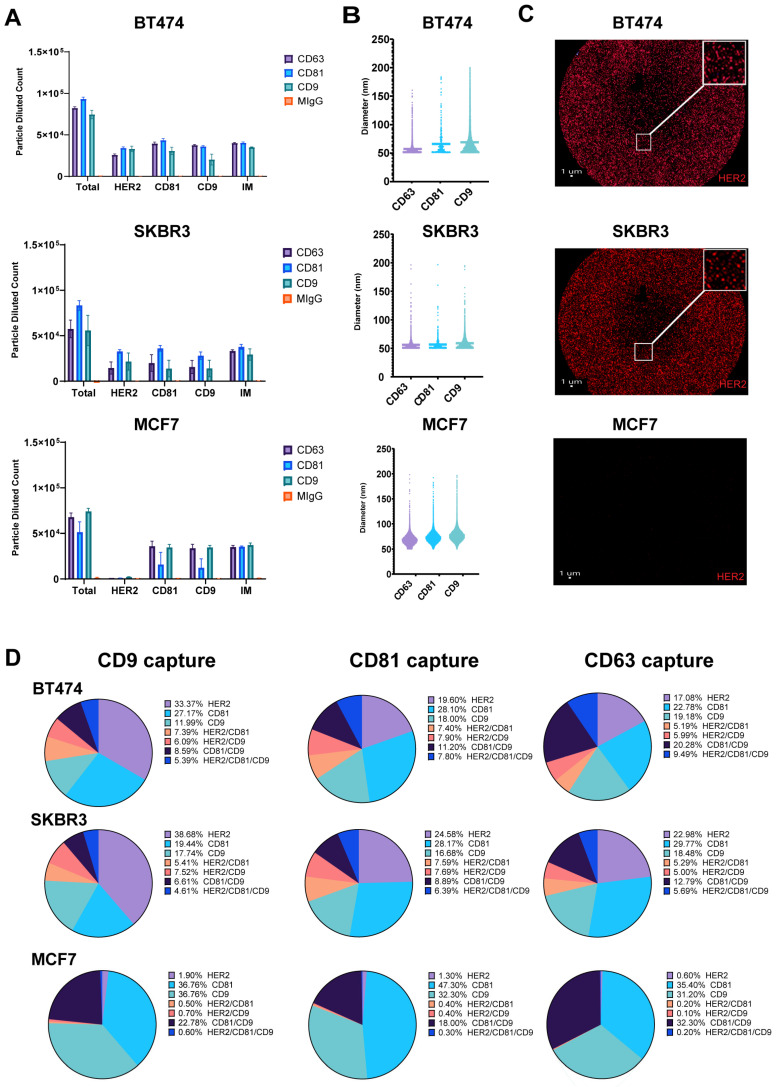
Detection of HER2-Positive Particles from Breast Cancer Cell Lines Using SP-IRIS. Exosomes were captured using tetraspanin-specific antibodies against CD9, CD81 and CD63 on the SPA chip and analyzed for HER2, CD9, and CD81 using fluorescent detection antibodies. (**A**) Total fluorescent particle counts of breast cancer-derived exosomes captured on a tetraspanin chip, with the x-axis representing the fluorescent detection markers (HER2, CD9, and CD81) and the interferometric particles (IMs). Y-axis represents the total particle count. Bars correspond to the antibody captures coated on the chip (Isotype mIgG-orange, CD9-aqua, CD81-blue and CD63-purple) and are presented with SD in triplicate. BT474 (**top**), SKBR3 (**middle**) and MCF7 (**bottom**). (**B**) Particle size distribution of EVs across each capture site for the three breast cancer cell lines, with bars representing the mean particle diameter. BT474 (**top**), SKBR3 (**middle**) and MCF7 (**bottom**). (**C**) CD9 capture spot montage. BT474 (**top**), SKBR3 (**middle**) and MCF7 (**bottom**). The red dots indicate a positive signal for the commercial HER2 detection antibody. (**D**) Colocalization of HER2, CD81, and CD9 across EVs derived from the three breast cancer cell lines, analyzed using three fluorescent channels and displayed as an overlay of fluorescent images across all Tetraspanin capture antibodies (CD9, CD81, CD63).

Breast cancer cell lines exhibit differential HER2 expression, influencing EV content and its role in intracellular communication. To characterize the molecular profiles of exosomes derived from HER2-high (BT474, SKBR3) and HER2-low (MCF7) expressing cell lines, we focused on HER2 and tetraspanin (CD9, CD81) composition, to uncover potential heterogeneity in exosome marker expression [27]. Exosomes purified from conditioned cell culture media were captured on Tetraspanin (CD9, CD81, CD63)-specific SP-IRIS chips and labeled with fluorescent detection antibodies against HER2, CD9, and CD81. Our analysis of exosomes derived from HER2-high (BT474, SKBR3) and HER2-low (MCF7) expressing breast cancer cell lines using SP-IRIS revealed distinct patterns in tetraspanin capture and HER2 expression; however, as expected, there are very few or no particles captured to the isotype control mIgG. In addition, the number of particles captured on all the tetraspanin markers for every cell line is consistent, proving the fact that the particles are exosomes (Figure 1A). CD81 captured the most total particles for SKBR3 and BT474 exosomes, while CD9 captured the most for MCF7 exosomes. SKBR3 and BT474 exosomes showed a two-fold increase in HER2-positive particles compared to MCF7 exosomes across all tetraspanin captures. Exosome size varied across cell lines and tetraspanin captures (Figure 1B), with MCF7 exosomes consistently larger (68.25–77.09 nm) compared to BT474 (57.52–69.69 nm) and SKBR3 (56.05–58.69 nm) exosomes. Analysis of CD9 capture spots (Figure 1C) showed higher HER2 expression in BT474 (33.37%) and SKBR3 (38.6%) exosomes compared to MCF7 (1.9%). These results suggest potential differences in exosome size and tetraspanin association among the three cell lines, with MCF7 exosomes displaying a broader size and a tendency toward larger vesicle populations. The analysis of CD9 capture spots revealed distinct HER2 and tetraspanin expression profiles across the three breast cancer cell lines. BT474-derived exosomes exhibited the highest surface HER2 expression at 33.37 ± 3.6%, followed by CD81 at 27.17 ± 3.8%, and CD9 at 12 ± 6.2%. Colocalization analysis for BT474 showed 7.4 ± 1.4% of exosomes positive for HER2/CD81, 6.1 ± 3.5% for HER2/CD9, 8.6 ± 4.9% for CD81/CD9, and 5.4 ± 3.3% triple-positive for HER2/CD81/CD9. SKBR3-derived exosomes displayed slightly higher HER2 expression at 38.6 ± 25.3%, while CD81 and CD9 were less prominent at 19.4 ± 17.2% and 17.7 ± 15.2%, respectively. SKBR3 colocalization patterns revealed 5.4 ± 7.2% HER2/CD81, 7.5 ± 9.1% HER2/CD9, 6.6 ± 9.6% CD81/CD9, and 4.6 ± 7.3% triple-positive exosomes. In contrast, MCF7-derived exosomes showed minimal HER2 expression at 1.9 ± 0.4%, with a stronger presence of both CD81 and CD9 at 36.8 ± 4.9% each. MCF7 exosomes exhibited the highest CD81/CD9 colocalization at 22.8 ± 5.1%, while HER2/CD81 (0.5 ± 0.2%), HER2/CD9 (0.7 ± 0.2%), and triple-positive exosomes (0.6 ± 0.2%) were rare (Figure 1D). These results report the differences in HER2 and tetraspanin expression relative values between HER2-high (BT474, SKBR3) and HER2-low (MCF7) breast cancer cell lines, as well as the varying degrees of marker colocalization on their respective exosomes.

### 2.2. Characterization of HER2-Positive Exosomes Captured by Trastuzumab Biosimilars

**Figure 2:**
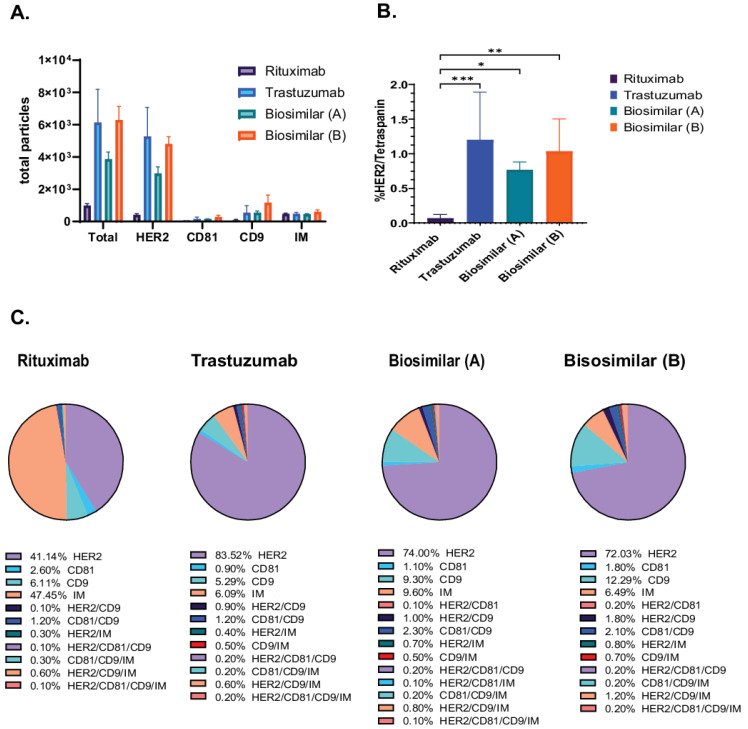
Characterization of HER2-Positive Exosomes Captured by Trastuzumab Biosimilars. Exosomes were captured using rituximab, trastuzumab, and biosimilar-specific conjugates in the Exo-Flex assay, with each capture performed in triplicate. (**A**) Total particle counts for rituximab, trastuzumab, and biosimilar captures [biosimilar A (Kanjiniti) and biosimilar B (Trazimera)], with the x-axis representing the detection antibodies and the y-axis showing the total particle count. Bars represent the mean particle counts, and error bars indicate. (**B**) Quantification of HER2/Tetraspanin is the sum of all HER/CD9/IM and HER2/CD9/CD81/IM particles, represented on the y-axis. Bars include SD, and statistical differences in binding affinity between trastuzumab, biosimilars, and rituximab captures were evaluated using a two-way ANOVA, with *p*-values considered highly significant (* *p*-value < 0.05; ** *p*-value < 0.01 and *** *p*-value < 0.001). Results are from three separate experiments, each done in triplicate. (**C**) Pie charts of colocalization analysis show the distribution of marker colocalization across conjugated antibodies, indicating the co-expression of these markers on bound particles. Data are presented as the mean of three replicates.

We employed the SP-IRIS-based methodology to evaluate the exosomal binding affinity of trastuzumab (Herceptin) and its biosimilars [biosimilar A (Kanjiniti) and biosimilar B (Trazimera)] in comparison to the non-HER2-targeted antibody rituximab (Rituxan), an anti-CD20 chimeric monoclonal antibody, using the Exo-Flex assay. Rituximab is utilized as a control antibody due to its identical Fc-region to that of trastuzumab. Additionally, given that both antibodies are employed in tumor therapy, we determined it to be the most appropriate background control for evaluating biosimilars and patient plasma samples. Exosomes derived from BT474 supernatants were purified and incubated with antibody-conjugated Exo-Flex chips. Following overnight binding and subsequent washing, captured exosomes were subjected on to the ExoView R100 platform using HER2, CD81, and CD9 detection antibodies for single-particle profiling and analyzed using Exoview Suite version 3.2.1. The data revealed that trastuzumab and its corresponding biosimilars captured 5.4× greater number of particles relative to the control antibody rituximab (anti-CD20 chimeric monoclonal antibody) (Figure 2A). In addition, detailed colocalization analysis revealed 17× greater number of HER2/Tetraspanin particles in all trastuzumab and biosimilar captures compared to the negative control capture with rituximab (Figure 2B). Furthermore, we identified distinct colocalization expression profiles across the four antibody capture conditions (Figure 2C). Trastuzumab-captured exosomes exhibited the highest HER2 expression at 83.6 ± 27.2%, followed by biosimilar A at 74.0 ± 10.2%, and biosimilar B at 72.1 ± 5.2%, while rituximab-captured exosomes showed significantly lower HER2 expression at 41.1 ± 6.6%. CD9 expression demonstrated relatively consistent levels across capture antibodies, with biosimilar B showing the highest expression at 12.3 ± 5.1%, followed by biosimilar A at 9.3 ± 1.5%, rituximab at 6.1 ± 3.8%, and trastuzumab at 5.3 ± 3.1%. In contrast, CD81 expression remained low across all conditions, with rituximab-captured exosomes displaying the highest levels at 2.6 ± 0.9%, while trastuzumab, biosimilar A, and biosimilar B showed minimal expression at 0.9 ± 0.5%, 1.1 ± 0.3%, and 1.8 ± 0.2%, respectively (Figure 2C). These results demonstrate the differential capture efficiency and surface marker expression patterns between HER2-targeting antibodies (trastuzumab and biosimilars) and the control antibody (rituximab) on BT474-derived exosomes.

### 2.3. Analysis of HER2-Positive Exosomes in Breast Cancer and Healthy Donor Plasma

**Figure 3:**
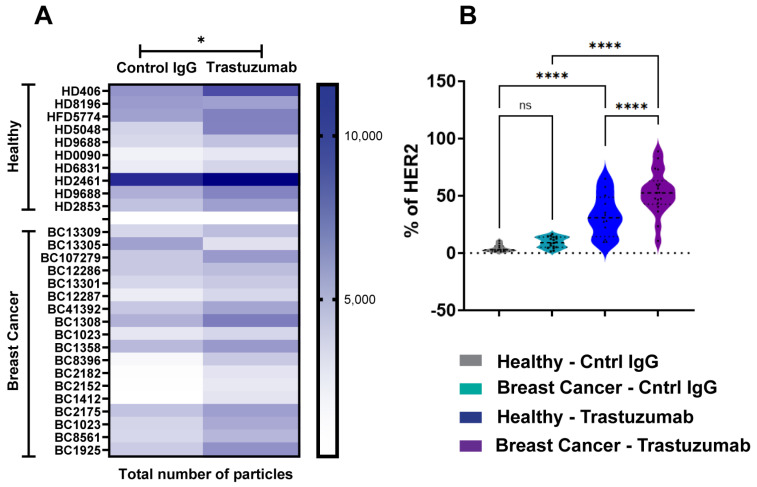
Analysis of HER2-Positive Exosomes in Breast Cancer and Healthy Donor Plasma. Exosomes were analyzed from healthy donors (*n* = 10) and breast cancer donors (*n* = 18) using an Exo-Flex chip conjugated with control IgG (rituximab) and trastuzumab. (**A**) Heatmap comparing EV binding across donors for each capture condition. Darker colors indicate increased binding, with statistically significant differences determined by a parametric *t*-test * *p* < 0.05. (**B**) Violin plot showing the percentage of HER2 detected on EVs for each capture condition. X-axis includes the following groups: healthy donor EVs captured on control IgG (grey), breast cancer donor EVs captured on control IgG (aqua), healthy donor EVs captured on trastuzumab (blue), and breast cancer donor EVs captured on trastuzumab (purple). Statistical differences were evaluated using a one-way ANOVA, with **** *p* < 0.0001 considered highly significant. Data are presented as the mean of three replicates. ns = non significant.

To evaluate SP-IRIS’s capability for biomarker identification, we analyzed HER2-positive exosomes purified from plasma samples derived from breast cancer (BC) patients and healthy donors (HDs). Using a tetraspanin Exo-Flex Chip (Fisher Scientific, Waltham, MA, USA) conjugated with trastuzumab and rituximab (control IgG-anti-CD20 chimeric monoclonal antibody), we observed that exosomes from BC samples exhibited significantly higher binding to trastuzumab-conjugated capture spots compared to rituximab spots (Figure 3A). This observation was consistent across multiple BC samples, indicating a specific enrichment of HER2-positive exosomes in the breast cancer cohort. While exosomes from HD samples also demonstrated preferential binding to trastuzumab-conjugated spots, the total number of HER2-positive exosomes captured was lower than in BC samples (Figure 3B). Quantitative analysis showed a 2-fold increase in HER2-positive exosomes in BC samples compared to HD samples (*p* < 0.001). These findings highlight the specificity of trastuzumab for capturing HER2-associated exosomes and demonstrate a notable difference in HER2-positive exosome expression between diseased and healthy cohorts. The differential capture of HER2-positive exosomes between BC and HD samples suggests that SP-IRIS could potentially serve as a sensitive method for detecting elevated levels of HER2-positive exosomes in plasma, which may correlate with HER2-positive breast cancer status. Our results underscore SP-IRIS’s capability to detect and quantify biomarker-specific exosomes in plasma samples, potentially distinguishing disease states based on exosomal biomarker profiles and opening avenues for non-invasive cancer diagnostics and monitoring.

### 2.4. Detection of Exosomal Immune Complexes Using SP-IRIS and Flow Cytometry

**Figure 4:**
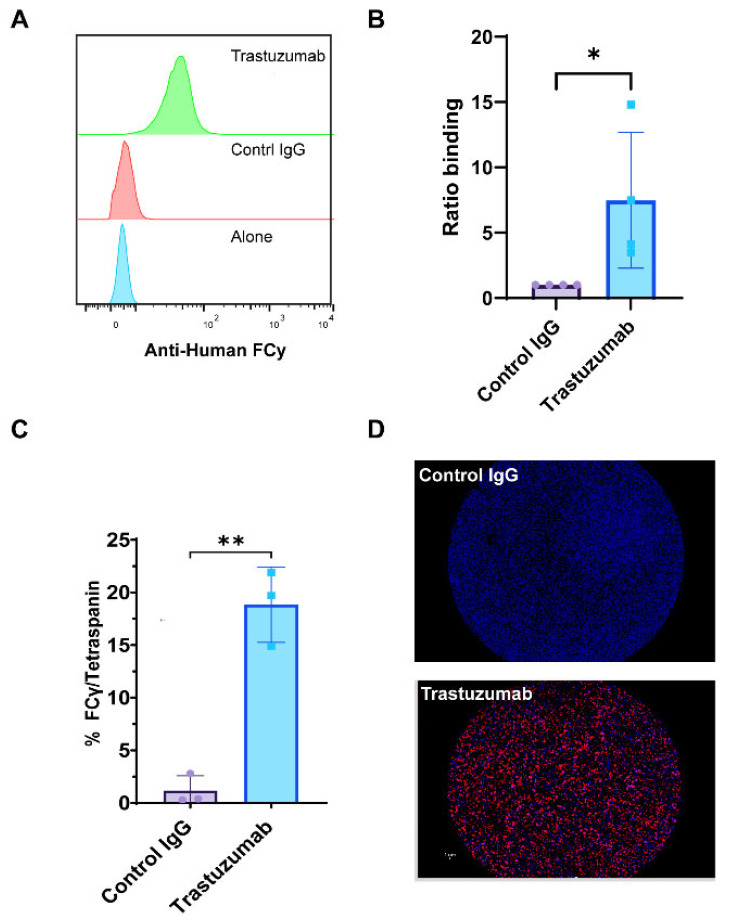
Detection of Exosomal Immune Complexes Using SP-IRIS and Flow Cytometry. Exosomes were analyzed using the SP-IRIS tetraspanin capture platform and a CD9 bead assay to evaluate immune complexes after overnight incubation with therapeutic antibody. (**A**) Relative histograms of anti-human Fcγ fluorescence intensities illustrate the distribution of Fcγ binding across the exosome populations. (**B**) The ratio of binding for anti-human Fcγ detected in the CD9 bead assay, normalized to the control IgG incubated exosome complexes, quantifying anti-human Fcγ binding. Results are from four separate experiments done from different exosome preps. Statistically significant results were determined by a parametric *t*-test (* *p* < 0.05). (**C**) Total particle counts of triple-positive particles detected for Fcγ/Tetraspanin using the SP-IRIS platform on a CD9 capture. Results are from three separate experiments of different exosome preps, all in triplicate. Statistically significant results were determined by a parametric *t*-test (** *p* < 0.01). (**D**) A representative spot montage of CD9 capture showing the colocalization of particles bound to the chip, with red indicating Anti-Human Fcγ, blue indicating CD9, and green indicating CD81.

We utilized SP-IRIS and CD9 bead flow cytometry assays to provide compelling evidence for the formation of HER2-positive immune complexes between trastuzumab and exosomes derived from BT474 breast cancer cells. Flow cytometry analysis, which quantifies the fluorescence intensity of large numbers of individual particles, revealed a significant increase in the mean fluorescence intensity (MFI) of anti-human Fcγ staining in trastuzumab-treated samples compared to control IgG (anti-βGal Human IgG) (Figure 4A,B). This increased MFI indicates a greater amount of antibody binding to the exosomes, suggesting successful immune complex formation. Complementing these findings, SP-IRIS analysis using CD9 capture chips showed a significantly higher number of HER2/tetraspanin particles in trastuzumab-treated samples compared to control IgG (anti-βGal Human IgG) (*p* = 0.028) (Figure 4C). The SP-IRIS technique, which allows for high-resolution imaging and quantification of individual nanoparticles, provided further confirmation of the immune complex formation at a single-particle level. Visual confirmation was provided by CD9 capture spot montages from SP-IRIS analysis, which revealed a higher density of anti-human Fcγ-positive particles in the trastuzumab-treated group (Figure 4D), offering a visual representation of the increased immune complex formation. These results collectively demonstrate that exosome-immune complexes can be effectively evaluated using both CD9-based flow cytometry and SP-IRIS methods, providing robust and multi-faceted evidence for the formation of HER2-positive immune complexes between trastuzumab and BT474-derived exosomes. The consistency between these two independent measurement methodologies strengthens the reliability of our findings and underscores the potential of these techniques for studying exosome-antibody interactions in various biomedical applications.

### 2.5. Impact of HER2-Positive Exosomes on Trastuzumab Binding and Immune Function

**Figure 5:**
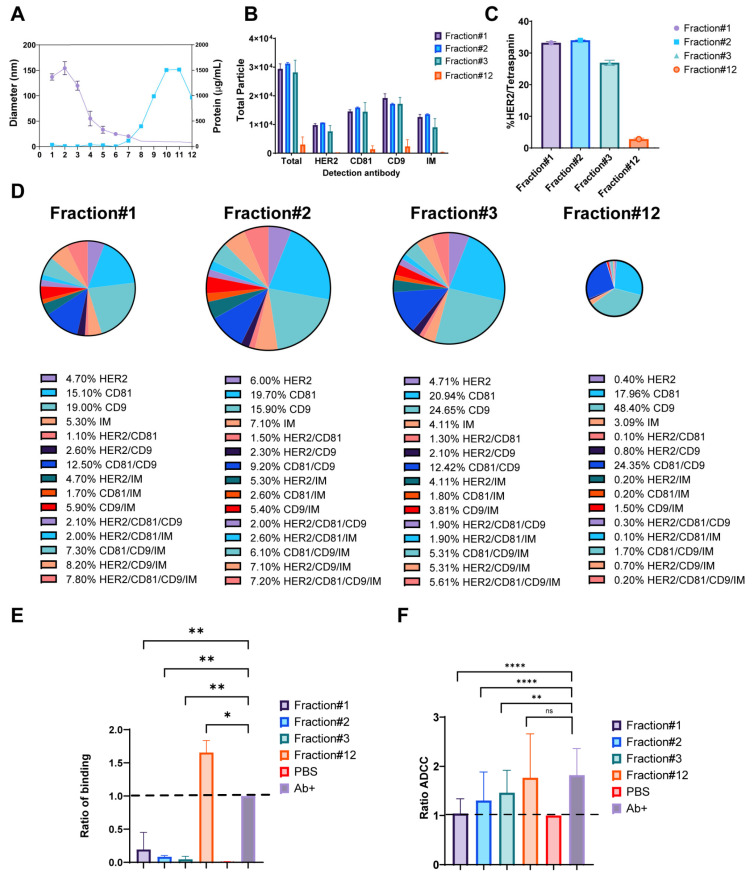
Impact of HER2-Positive Exosomes on Trastuzumab Binding and Immune Function. (**A**) Particle diameter on the left y-axis (purple) and protein concentration (µg/mL) on the right y-axis (blue), with the x-axis representing fractions 1–12. (**B**) Total fluorescent particle counts of fractions 1–3 and 12 captured on a CD9 capture, with the x-axis representing the fluorescent detection markers (HER2, CD9, and CD81) and the y-axis representing the total particle count. Bars correspond to the individual fractions incubated on the chip and are presented with SD in triplicate. (**C**) Quantification of HER/Tetraspanin particles captured by CD9 expressed as a percentage. Bars represent the SD from an experiment done in triplicate. (**D**) Colocalization analysis for EVs captured on CD9 from fractions 1–3 and 12. (**E**) Column graph depicting the ratio of binding for fractions 1–3 and 12, normalized to the antibody alone control indicated by a black-dashed line. The y-axis represents the ratio of binding, calculated as the signal from the anti-human Fcγ label divided by the signal from the antibody alone control. Data are presented as mean values with SD bars, representing *n* = 2 separate exosome preparations. Statistical analysis was performed using a one-way ANOVA, with Dunnett’s multiple comparisons test, ns (not significant), * *p* < 0.05 and ** *p*-value < 0.01, considered significant. (**F**) Column graph showing ADCC ratios for fractions 1–3 and 12, normalized to the DPBS control. Data are presented as mean values with SD error bars. Statistical analysis was performed using a two-way ANOVA, ** *p* < 0.01 and **** *p* < 0.0001 (*n* = 4 donors). ns = non significant.

With a confirmation that trastuzumab can form exosomal-immune complexes in vitro, functional assays were performed to assess the impact of these complexes on trastuzumab’s immune-mediated mechanisms of action. Results from size exclusion chromatography fractionation of BT474-derived exosomes revealed distinct characteristics across fractions. Early fractions (1–3) contained peak exosome-sized particles ranging from 100–150 nm in diameter, as determined by Dynamic Light Scattering (DLS) (Fisher Scientific, Waltham, MA, USA), while later fractions showed increased protein levels (Figure 5A). Particle enumeration using SP-IRIS analysis provided insights into the distribution and characteristics of exosomes across fractions. Notably, the total number of particles bound to CD9-capture spots was predominantly enhanced in Fractions 1–3, indicating a high concentration of exosomes in these early fractions. In contrast, Fraction 12 exhibited minimal particle binding and was subsequently used as a negative control for functional studies (Figure 5B). The comprehensive marker analysis of BT474-derived exosomes revealed intricate patterns of protein expression and colocalization. Overall, 28–33% of particles in early fractions (1–3) co-expressed HER2 and tetraspanin markers, which was greater than the 2.5% observed in Fraction 12 (negative control) (Figure 5C). Analysis of exosome fractionation revealed distinct marker expression patterns across the gradient fractions. Early fractions (1–3) demonstrated substantially higher individual marker expression, with CD9 showing the most pronounced variation from 19.0 ± 1.0% in Fraction 1 to 24.6 ± 3.0% in Fraction 3, followed by CD81 ranging from 15.1 ± 0.3% to 20.9 ± 2.7%, and HER2 maintaining relatively consistent levels from 4.7 ± 0.3% to 6.0 ± 0.4%. In contrast, late fractions showed dramatically reduced expression, with Fraction 12 displaying minimal levels of HER2 (0.4 ± 0.1%), CD81 (18.0 ± 8.3%), and CD9 (48.5 ± 42.9%). Colocalization analysis revealed complex marker combinations predominantly in early fractions. CD81/CD9 double-positive particles were most abundant, ranging from 9.2 ± 0.2% in Fraction 2 to 12.5 ± 0.9% in Fraction 1, while increasing substantially in later fractions to 24.4 ± 29.0% in Fraction 12. HER2-containing combinations showed distinct patterns: HER2/CD9/IM triple-positive particles peaked at 8.2 ± 1.5% in Fraction 1, declining to 0.7 ± 1.1% in Fraction 12, while HER2/CD81/CD9/IM quadruple-positive particles followed a similar trend from 7.8 ± 0.1% to 0.2 ± 0.3%. HER2/CD9 double-positive particles remained relatively stable across early fractions (2.1 ± 0.2% to 2.6 ± 0.5%) before decreasing in later fractions (Figure 5D). These results demonstrate the heterogeneous nature of exosome populations, with early fractions enriched in HER2-positive, multi-marker exosomes, while later fractions contain predominantly tetraspanin-positive particles with minimal HER2 expression. The functional analysis of exosome-trastuzumab interactions provided critical insights into the potential impact of tumor-derived exosomes on antibody-based therapies. Exosomes were incubated overnight with 5 µg/mL trastuzumab to form immune complexes, followed by ultracentrifugation to separate bound and unbound antibodies. Two key assays were employed to assess the effects of these interactions. First, the cell-binding assay revealed that supernatants obtained after the formation of trastuzumab-exosome immune complexes, particularly from early fractions, inhibited trastuzumab binding to HER2-positive BT474 cells (Figure 5E). This inhibition suggests that exosomes can effectively sequester trastuzumab, reducing availability to bind to target cells. Intriguingly, supernatant from Fraction 12 enhanced trastuzumab binding to BT474 cells, although the mechanism behind this enhancement remains unclear. Second, the antibody-dependent cell-mediated cytotoxicity (ADCC) assay showed that trastuzumab’s ADCC activity was decreased when incubated with exosomes from early fractions (Figure 5F). This reduction in ADCC activity highlights the capability of exosomes to sequester trastuzumab and impair the mAb immune effector functions. Notably, the enhanced binding observed with Fraction 12 did not correlate with increased ADCC activity, suggesting that binding alone was not sufficient to predict functional outcomes. These findings collectively demonstrate that HER2-enriched exosomes from early fractions can effectively sequester trastuzumab and reduce its immune effector functions, potentially acting as decoys for antibody therapeutics.

## 3. Discussion

The complex interplay between exosomes and therapeutic antibodies in breast cancer presents both challenges and opportunities for improving treatment strategies. Our study provides insights into this relationship, particularly focusing on HER2-positive exosomes and their interactions with trastuzumab or its biosimilars. This study also provides an analysis of HER2-positive exosomes in breast cancer, utilizing Single-Particle Interferometric Reflectance Imaging Sensor (SP-IRIS) technology alongside functional assays. Our SP-IRIS analysis of exosomes derived from breast cancer cell lines revealed distinct patterns of HER2 and tetraspanin expression, contributing to the growing body of knowledge on exosome heterogeneity and their potential as biomarkers. This aligns with the broader field of extracellular vesicle (EV) research, which has seen advancements in recent years. The heterogeneity we observed in exosome populations, particularly in terms of HER2 and tetraspanin expression, was consistent with findings from other studies, which demonstrated that different subpopulations of EVs can be distinguished based on their size and protein composition, emphasizing the importance of comprehensive characterization [26,28]. Our results, showing variations in HER2 and tetraspanin expression across different breast cancer cell lines, support this concept of exosome heterogeneity. The predominance of CD81 in HER2-overexpressing exosomes (BT474, SKBR3) that we observed aligns with previous work, which highlighted the role of tetraspanins in exosome biogenesis and cargo selection [29]. Furthermore, our findings on the co-expression of HER2 with tetraspanins on exosomes are supported by studies that demonstrated that HER2 could be actively sorted into exosomes in breast cancer cells [30]. The larger size of MCF7-derived vesicles may indicate alternative biogenesis mechanisms in luminal A breast cancer, highlighting the heterogeneity of exosome populations across different breast cancer subtypes [31]. Exo-Flex assays provided insights into the binding affinities of trastuzumab or its biosimilars to HER2-positive exosomes. Although not significant, the observation that trastuzumab captured more HER2/Tetraspanin particles than its respective biosimilars is intriguing. This discrepancy could be attributed to differential affinities for membrane-bound HER2 on vesicles versus soluble HER2 in the tumor microenvironment [15]. Such differences underscore the importance of adequate characterization when evaluating biosimilars and highlight the potential use of SP-IRIS for regulatory applications. The greater levels of HER2-positive exosomes in breast cancer patient plasma compared to healthy donors likely reflect tumor-derived vesicle overexpression. The overexpression finding aligns with previous studies showing elevated levels of tumor-derived exosomes in cancer patients [1,32]. The presence of HER2-positive exosomes in healthy donors, albeit at lower levels, suggests a potential physiological role for these vesicles in normal cellular communication or immune regulation [33]. The potential of exosomes as cancer biomarkers has gained attention in recent years, with our study contributing to this growing field by demonstrating the ability to detect HER2-positive exosomes in plasma samples from breast cancer patients using SP-IRIS technology. This approach aligns with similar advancements in other cancer types, such as the identification of glypican-1-positive exosomes for early detection of pancreatic cancer [34], and the development of a urine exosome gene expression assay for high-grade prostate cancer detection [35], along with the use of exosomal microRNAs as diagnostic and prognostic biomarkers in ovarian cancer [36]. Our SP-IRIS method offers enhanced sensitivity and specificity compared to these studies, addressing limitations of current clinical methods like FISH and IHC, which are known for their invasiveness and potential for diagnostic errors in HER2 detection [37]. This advancement of the SP-IRIS approach would address the need for more precise and comprehensive exosome characterization methods, as highlighted by the International Society for Extracellular Vesicles (ISEVs) in their minimal information for studies of extracellular vesicles (MISEVs) guidelines [38].

Perhaps the most clinically relevant finding of our study is the demonstration that HER2-positive exosomes can act as decoys, sequestering trastuzumab and potentially reducing its efficacy in activation of antibody-dependent cellular cytotoxicity (ADCC). This ADCC-related phenomenon was most pronounced in early Size exclusion chromatography (SEC) fractions enriched with HER2-positive exosomes. These results provide a potential mechanistic explanation for the variable response to trastuzumab observed in clinical settings and suggest that exosomal HER2 levels could serve as a predictive biomarker for treatment efficacy. In line with our observations, a study published in Nature demonstrated that higher levels of circulating exosomal PD-L1 are associated with a poorer response to anti-PD-1 therapy [24]. This research highlighted the role of exosomes as potential decoys in the tumor microenvironment (TME), suggesting that these vesicles can mimic target cells and divert antibody binding away from intended targets [39]. This finding underscores the importance of considering exosomal factors in predicting and understanding immunotherapy responses, and it supports the concept that exosomes may play a significant role in modulating the efficacy of antibody-based cancer treatments.

We acknowledge several limitations in this study. In vitro experiments may not fully replicate the complexity of the tumor microenvironment. Additionally, the number and diversity of clinical samples remain limited. Expanding to larger cohorts and different stages of breast cancer would help validate SP-IRIS as a broadly applicable diagnostic tool. With these caveats in mind, routine measurements of HER2-positive exosomes could inform therapeutic decisions, such as adjusting trastuzumab dosing or changing treatment strategies upon early signs of resistance. Targeting exosomes, perhaps by inhibiting their release or uptake, might preserve monoclonal antibody efficacy in resistant tumors. Incorporating SP-IRIS into larger clinical trials could further establish a role for this technology in therapy monitoring and potentially lead to the identification of novel biomarkers.

## 4. Materials and Methods

### 4.1. Cell Culture

BT474, SKBR3 and MCF7 breast cancer cells were procured from ATCC (Manassas, VA, USA) and cultured in RPMI, DMEM+F12 and DMEM, respectively; supplemented with 10% fetal bovine serum (FBS), 100 U/mL PenStrep, 2 mM GlutaMax, 1× MEM Non-Essential Amino Acids, and 1 mM sodium pyruvate (Invitrogen, Thermo Fisher Scientific, Waltham, MA, USA). The cells were primarily grown in 100 mm Petri dishes or tissue culture-treated T75 flasks, depending on the initial seeding density. For the purification of exosomes, the cells were cultured with complete media containing 10% exosome-depleted FBS at 75–80% confluency.

### 4.2. Purification of Exosomes

After 48 h of incubation with exosome-depleted media, the conditioned medium was harvested and subjected to a series of centrifugation steps to remove any remaining cellular debris. The supernatant was then clarified either using a 0.22 µm or 0.45 µm filter and concentrated ten times using a 100 kDa MWCO filter before proceeding to downstream EV purification. Exosomes were purified using several different techniques: (1) Ultracentrifugation: Supernatants were centrifuged at 100,000× *g* for 1 h, washed once with DPBS with an additional centrifugation step for one hour, and the pellet was reconstituted in 1× DPBS. (2) Precipitation: Using the Total Exosome Isolation Reagent specific for cell culture media kit from Invitrogen (Catalog #4478359, Thermo Fisher Scientific, Waltham, MA, USA), the concentrated supernatants were incubated overnight with the reagent, and the exosomes were precipitated according to the manufacturer’s protocol. (3) Size exclusion chromatography (SEC): QEV 70 nm columns from IZON Biosciences (Medford, MA, USA) were used with the Automatic Fraction Collector to collect fractions of the supernatants, and peak fractions containing exosomes were either pooled or left analyzed as single fractions.

### 4.3. Analysis of Exosomes

Exosomes were characterized using three analytical techniques; each applied thoroughly and precisely: Dynamic Light Scattering (DLS), Flow cytometry, and Single-Particle Interferometric Reflectance Imaging Sensor SP-IRIS. DLS: 40 µL of each diluted exosome sample was transferred to a Greiner Bio-One 384-well plate (Cat# P8803-384) and analyzed using a Wyatt DynaPro plate reader (Wyatt Technology LLC, Santa Barbara, CA, USA). Flow cytometry: Analysis of the exosomes was conducted using Invitrogen Dynabeads CD9 beads (Cat# 10614D, Fisher Scientific, Waltham, MA, USA) following the manufacturer’s protocol. We combined exosome samples with CD9-coated Dynabeads in a 1:20 ratio (sample to beads) and incubated them overnight at 4 °C with gentle rotation to promote optimal binding. Post-incubation, the beads were thoroughly washed in DPBS containing 0.1% BSA to minimize non-specific binding, according to the manufacturer’s wash guidelines. After the wash step, samples were stained with a fluorescently labeled antibody specific to anti-human IgG, Fcγ fragment before acquiring the samples on the BD LSRFortessaX-20 cell analyzer. SP-IRIS: Exosome characterization was performed using the Leprechaun Exosome Human Tetraspanin Flex kits (Part# 215-1057) or the Leprechaun Exosome Human Tetraspanin kits (without Flex captures, Part#251-1044) according to the manufacturer’s protocols (Unchained Labs LLC, Pleasanton, CA, USA). The tetraspanin markers coated on the chips are antibodies against CD9, CD81 and CD63, along with respective isotype control mouse IgG (mIgG). For the flex experiments, desired antibodies [control IgG (anti-β-Gal-hIgG1), rituximab (anti-CD20 chimeric monoclonal antibody), trastuzumab (anti-human HER2) and biosimilars (anti-human HER2)] were buffer exchanged to a final 0.5 mg/mL concentration, which was essential to stabilize the antibodies, ensuring compatibility with the linker conjugation reagents. Following buffer exchange, antibody-linker conjugation was performed by incubating the antibodies with a proprietary linker solution per the manufacturer’s instructions, yielding stable antibody-linker complexes optimized for chip binding. The conjugated antibodies were then applied to Tetraspanin Flex SP-IRIS chips in a 30 min pre-coating step, allowing efficient immobilization of the antibody-linker complexes on the chip surface. After coating, the chips were washed using the Flex Prep program on the Unchained Labs Leprechaun chip washer (Unchained Labs LLC, Pleasanton, CA, USA) to eliminate unbound antibodies, ensuring a specific surface for exosome capture. Once prepared, exosome samples were incubated on the antibody-coated chips overnight at room temperature, allowing binding interactions between the exosomes and the surface-immobilized antibodies. The next day, post-incubation washes were performed using the Exosome protocol on the Unchained Labs chip washer to remove unbound material. The chips were then stained with surface marker antibodies to facilitate downstream analysis. Finally, samples were analyzed using the ExoView R100, providing quantitative and qualitative data on exosome capture and specific surface marker expression. The detection markers vary depending on the purpose of the experiment. The specific fluorescent antibodies used in the experiment were detailed in the figure legends of that section. This method enabled sensitive and reproducible detection of exosome populations, tailored through targeted antibody conjugation and controlled chip preparation protocols.

### 4.4. Healthy Donor and Breast Cancer Plasma Exo-Flex Assay

Plasma samples of breast cancer patients and healthy donors were procured under Institutional Review Board (IRB)-approved protocol from commercial vendors Cureline, Inc. (South San Francisco, CA, USA). and Discovery Life Sciences, Inc. (Huntsville, AL, USA). Plasma was centrifuged at 10,000× *g* for 20 min to remove cellular debris. Purification was performed using the Invitrogen Total Exosome Isolation Reagent per the manufacturer’s protocol without proteinase treatment. Briefly, diluted plasma samples were incubated overnight at 4 °C with the exosome isolation reagent, followed by centrifugation and the final exosome pellet was resuspended in 50 µL of 1× DPBS buffer. Purified exosomes were analyzed using an Exo-Flex chip conjugated with trastuzumab and rituximab capture. Exosome samples were diluted 1:5 and incubated on the flex-chip to allow for visualization. Detection was performed using commercial HER2 APC, CD9 PE, and CD81 FITC antibodies.

### 4.5. Immune Complexes and Antibody Depletion

Using the SEC method of purification, exosomes were purified, and the respective exosome-immune complexes (Exo-IgG) were prepared using trastuzumab and biosimilar antibody products. Briefly, exosomes were incubated with a 1:2 dilution of 5–10 µg/mL trastuzumab in incubation buffer overnight at 4 °C on a Hula Mixer (Thermo Fisher Scientific, Waltham, MA, USA). The next day, the Exo-IgG complexes were subjected to ultracentrifugation at 100,000× *g* for 1 hour at 4 °C. After the first round of ultracentrifugation, 100 µL of the supernatant was collected. The complexes were washed once with 1× DPBS and resuspended in a final volume of 25 µL of 1× DPBS and then subjected to either a CD9 bead assay or SP-IRIS to detect the immune complexes. The collected supernatants were further used for the BT474 cell binding and ADCC assays.

### 4.6. BT474 Cell Binding Assay

After trypsinization, BT474 cells were transferred to sterile Falcon 12 × 75 mm, 5 mL polystyrene tubes (without caps) at a concentration of approximately 1 × 10^5^ cells per condition. The cells were washed once with FACS buffer (0.1% FBS in DPBS) and tested for HER2 expression either using fluorescent-labeled HER2 (BD APC Mouse Anti-Human HER-2/neu) or unlabeled trastuzumab followed by a secondary detection antibody (Jackson Labs APC Affinity Pure Goat Anti-Human IgG, Fcγ Fragment Specific). Subsequently, 2.5 µL (12.5 ng approx.) of the supernatants collected during the initial step of Exo-IgG complex formation were added to the cells as primary antibody incubation. Cells were washed and detected using a secondary detection antibody, Goat Anti-Human Fcγ APC. Following primary incubation, the cells were washed twice with FACS buffer. A secondary detection antibody, Goat Anti-Human Fcγ APC (Cat# 109-135-098 Jackson Immuno, West Grove, PA, USA), was added, and the cells were incubated on ice for 25 min. After incubation, the cells were washed twice and resuspended in 300 µL of FACS buffer. Data acquisition was performed on a BD LSR Fortessa X-20 SORP flow cytometer (BD Biosciences, San Jose, CA, USA).

### 4.7. Antibody-Dependent Cell-Mediated Cytotoxicity (ADCC)

Leukapheresis packs were obtained under an Institutional Review Board (IRB)-approved protocol from the National Institutes of Health (NIH) Blood Bank (Bethesda, MD, USA), with all donors providing informed consent. Peripheral blood mononuclear cells (PBMCs) were isolated using Ficoll-Paque™ Premium density gradient media (Cat# 17544203, Cytiva, Marlborough, MA, USA). NK cells were then purified from PBMCs using a negative selection kit (Cat# 17955, STEMCELL™ Technologies, Vancouver, BC, Canada). The purity of the NK cells was confirmed via flow cytometry using CD3 (clone UCHT1), CD56 (clone CMSSB), and CD16 (clone eBioCB16) surface markers (all antibodies from Invitrogen). Following purification, NK cells were stimulated with 25 U/mL recombinant human IL-2 (rIL-2; Teceleukin, NCI, Frederick, MD, USA) in Iscove’s Modified Dulbecco’s Medium (IMDM, Gibco, Thermo Fisher Scientific, Waltham, MA, USA), supplemented with 10% fetal bovine serum (FBS, Gibco), 2 mM L-glutamine (Gibco), 1 mM sodium pyruvate (Gibco), and 1× non-essential amino acids (Gibco). NK cells were incubated overnight to promote activation before performing antibody-dependent cellular cytotoxicity (ADCC) assays. For the ADCC assay, target cells were cultured in low-Ig serum media and prepared prior to the assay. The assay followed the manufacturer’s instructions (aCella™-TOX, Cat# CLATOX100-3L, Cell Technology, Fremont, CA, USA). Briefly, 5 × 10^3^ target cells per well were co-cultured with NK cells at 10:1 in ADCC culture media containing low-Ig serum, with or without trastuzumab (5 μg/mL). After 3–4 h of incubation, the GAPDH released into the supernatant was measured using bioluminescent reagents in the assay kit. The percentage of specific cell lysis was calculated using the following formula:

$$\%\mathrm{Cytotoxicity}\;=\;\frac{\begin{array}{c} Experimental\;release-Spontaneous\;release\;from\;target\;and\;effector\;cells\; \end{array}}{Maximum\;release-Spontaneous\;release\;from\;target\;cells\;alone}\;\times \;100$$

### 4.8. Statistical Methods

For column analysis, statistical comparisons were performed using a two-tailed parametric Student’s *t*-test to assess differences between specific groups. Additionally, a two-way analysis of variance (ANOVA) with multiple comparisons was conducted to analyze interactions and effects within the ADCC assay conditions. All statistical analyses were conducted under the assumption of normal distribution, and significance was determined at *p* < 0.05. Error bars in all graphs represent standard deviations (SDs), measuring data variability across replicate experiments. Statistical analyses were performed using GraphPad Prism v10 (Boston, MA, USA). Additionally, FlowJo v10, LLC (Ashland, OR, USA) was used to analyze the data obtained from the LSR-Fortessa X-20, BD Biosciences (Milpitas, CA, USA).

## 5. Conclusions

In conclusion, our study contributes to the growing field of exosome-based biomarkers in cancer, offering a new approach for HER2 detection and characterization. The SP-IRIS technology provided a potential tool for exosome analysis that may have clinical applications. As we continue to unravel the complexities of exosome biology, these findings may provide better strategies for breast cancer diagnosis and treatment.

## Data Availability

All the authors agree to share the raw data upon request.

## Abbreviations

The following abbreviations are used in this manuscript:

ADCC	Antibody-Dependent Cell-Mediated Cytotoxicity
ATCC	American Type Culture Collection
BC	Breast Cancer
BSA	Bovine Serum Albumin
DLS	Dynamic Light Scattering
DPBS	Dulbecco’s Phosphate Buffered Saline
EMT	Epithelial-to-Mesenchymal Transition
EV	Extracellular Vesicle
FACS	Fluorescence-Activated Cell Sorting
FBS	Fetal Bovine Serum
FISH	Fluorescence In Situ Hybridization
GAPDH	Glyceraldehyde 3-Phosphate Dehydrogenase
HD	Healthy Donor
HER2	Human Epidermal Growth Factor Receptor 2
IHC	Immunohistochemistry
IL-2	Interleukin-2
IMDM	Iscove’s Modified Dulbecco’s Medium
ISEV	International Society for Extracellular Vesicles
MAPK	Mitogen-Activated Protein Kinase
MEM	Minimum Essential Medium
MISEV	Minimal Information for Studies of Extracellular Vesicles
PBMCs	Peripheral Blood Mononuclear Cells
PD-L1	Programmed Death-Ligand 1
PI3K/AKT	Phosphoinositide 3-Kinase/Protein Kinase B
RAD51	DNA repair protein
rIL-2	Recombinant human Interleukin-2
RPMI	Roswell Park Memorial Institute (medium)
SEC	Size Exclusion Chromatography
SP-IRIS	Single-Particle Interferometric Reflectance Imaging Sensor
TDE	Tumor-Derived Exosomes
TME	Tumor Microenvironment
